# An Evaluation of Regional Cardiovascular Disease and Cancer Research Needs Using Conference Abstracts

**DOI:** 10.5334/aogh.2886

**Published:** 2021-08-05

**Authors:** Georgia A. Williamson, Shelly Rodrigo, Elizabeth Blackman, Camille C. Ragin, J. Robert Beck, Marshall K. Tulloch-Reid

**Affiliations:** 1 Caribbean Institute for Health Research (CAIHR), The University of the West Indies, Mona. Kingston, JM; 2 University of the West Indies, St. Augustine, Port of Spain, TT; 3 Fox Chase Cancer Center, Philadelphia, US; 4 African Caribbean Cancer Consortium (AC3), Philadelphia, US; 5 Professor of Epidemiology and Endocrinology, Epidemiology Research Unit, Caribbean Institute for Health Research (CAIHR), The University of the West Indies, Mona, 7 Ring Road, Mona Kingston 7, JM

## Abstract

**Background:**

Despite cardiovascular diseases and cancer being the leading causes of premature mortality in the Caribbean region, there is limited local research available to guide a comprehensive response to this epidemic.

**Objective:**

To evaluate cardiovascular disease and cancer research in the Caribbean using abstracts presented at the Caribbean Public Health Agency’s (CARPHA) meeting – the longest running annual research conference in the region.

**Method:**

Study data (population, intervention/exposure, comparison and outcome) were extracted from abstracts published for the 2006 to 2018 meetings. Additionally, institutional affiliation and geographic location of the first author, countries involved, sample size, study design and use of specialized testing/biomarkers were also extracted. Data were analysed using STATA version 14.

**Findings:**

A total of 1,512 abstracts, 728 posters and 784 oral presentations were reviewed. Research on cancer and cardiovascular disease comprised approximately 15% of all abstracts published annually over the review period. Most of the cardiovascular disease studies had cross sectional or survey designs (46%), with very few laboratory-based studies (<2%) and no intervention studies/clinical trials. For cancer research, 30% were cross-sectional studies/audits, 11% were case control studies, 5% were lab based and there were no clinical trials. Almost a quarter of the cardiovascular disease / cancer abstracts over the period originated from Trinidad and Tobago (26%), with Jamaica and Barbados contributing 18% and 15% respectively.

**Conclusion:**

These finding highlight the need for additional studies that can provide evidence for interventions and policy to address the region’s high cardiovascular disease and cancer burden. A Regional Centre of Research Excellence could support capacity development to facilitate this process.

## Introduction

Non-communicable diseases (NCD), including cancers, cardiovascular diseases (CVD), chronic respiratory diseases and diabetes, are the leading cause of mortality globally. These diseases contribute to 71% of all deaths globally, with cancers (22%) and cardiovascular diseases (44%) accounting for the majority of these deaths [1]. The Caribbean, consisting primarily of middle- and high-income small island developing states (SIDS), has one of the highest burdens of NCDs in the world (1), likely resulting from urbanization, an ageing population, lifestyle practices and inadequate access to health technologies [2]. The epidemiologic transition in disease patterns in the Caribbean started during the 1960s, with a shift in the majority of deaths resulting from malnutrition and infectious diseases in the 1950s to NCDs by the early 1970s [3]. Since the epidemiologic transition, CVDs (including hypertension, heart disease and stroke) has remained the leading cause of mortality in the Caribbean [1].

The Caribbean was one of the first regions to recognize the potential effect of NCDs on its nations’ economies and development, resulting in the Port of Spain Declaration [4]. The Declaration signed by the Caribbean Community (CARICOM) Heads of Government pledged to tackle the region’s NCD epidemic through a series of strategies and policies. This initiative led to the 2011 United Nations High Level Meeting on NCDs and helped to shape the global NCD agenda [5]. Despite this laudable achievement, the Caribbean has lagged in research initiatives to find solutions to the growing NCD burden [6]. It is against this background that we conducted a research needs assessment by evaluating regional research output in cancer and CVD using abstracts from the Caribbean Public Health Agency’s (CARPHA) annual scientific meeting.

Hosted annually since 1956, the CARPHA meeting is the largest health research conference in the English-speaking Caribbean. Initially a forum for presenting findings from investigators from the United Kingdom (UK) Medical Research Units based in the Caribbean (the Commonwealth Caribbean Medical Research Council Meeting), the CARPHA conference is now considered a driver of public health research and an avenue for the dissemination of new research being undertaken in the region. The CARPHA conference engages hundreds of researchers (conducting basic science, animal and human subject research), policy makers and healthcare providers in rigorous discussions that identify research priorities for strengthening national and regional health research systems [7].

This assessment focused on research abstracts presented at the CARPHA annual research meetings. The main aim was to 1) determine what types of research were being conducted in cancer and cardiovascular disease in the Caribbean; 2) identify the key stakeholders and institutions involved in the process; 3) and determine scientific quality and volume of research in these areas.

## Methodology

A review of conference abstracts published in the West Indian Medical Journal from 2006 to 2018 was conducted. The PICO model (population, intervention/exposure, comparison, outcome) for clinical and health research was used as the framework to extract information about the studies presented [8]. Additional details collected from each abstract included name and institutional affiliation of the first author, countries and institutions involved in the research, sample size, study design, use of specialized diagnostic test and biomarker measurements. Data from the abstracts were extracted into an Excel spreadsheet.

Two teams worked on the extraction of the data separately. At the end of the extraction for each year, the groups met for quality checks, which included verifications of abstract details, identifying and addressing discrepancies and making modification to the extraction tool. An Excel function field was utilized to create formulae for coding abstracts after creating an agreed upon classification developed by the extracting team. Medical subject headings (MESH) for cancer and cardiovascular disease gathered from the PubMed online search engine were used to identify key words to be used in coding the data. Coding allowed for identification of 1) research on cancer and CVD; 2) organizations involved in research (e.g., University, Ministry of Health, hospital, etc.); 3) country of origin for research; 4) research collaborations (e.g., in-country, regional, international, etc.). Data were transferred to the STATA software version 14 (College Park, TX) for analysis. In addition to the descriptive analysis of the abstracts reviewed, the concordance between stated study design and evaluation by the data extraction team was also examined. We compared reviewer determined study designs based on the abstract findings with those reported by authors, to assess whether there was agreement with what was presented.

## Results

One thousand five hundred and twelve (1,512) abstracts, comprising of 728 poster presentations and 784 oral presentations were reviewed (***Table 1***). Most of the research originated from Trinidad (29%), Jamaica (18%) and Barbados (15%). Among the types of institutions identified, universities produced the vast majority (74.1%) of the research, followed by Ministries of Health (8.7%) and hospitals (7.1%). Cancer and CVD represented approximately 7% and 9% respectively (approximately 15% combined) of the abstracts reviewed over the period. Most of the research (74.0%) was conducted in collaboration with regional institutions and almost a quarter (23.2%) represented collaborations with various international institutions. Blood (29%), urine (11%) and body fluids (4%) were the most common bio-specimens collected from these reports.

**Table 1:** Characteristics of Research papers accepted for the CARPHA conferences from 2006 to 2018 (1,512).


CATEGORIES	FREQUENCY (#)	PERCENT (%)

Presentations		

Orals	784	51.9

Posters	728	48.2

Country of Origin		

Trinidad	438	29.0

Jamaica	272	18.0

Barbados	227	15.0

Other	575	38.0

Institution Affiliation		

University	1120	74.1

Ministry of Health	132	8.7

Hospital	107	7.1

Other	153	10.1

Disease Areas		

Cancer	101	6.7

CVD	131	8.7

Other	1,270	84.0

Collaboration		

In-Country	1,067	74.3

Regional	40	2.8

International	327	22.8

Biomarkers Collected		

Blood	65	29.4

Urine	24	11.0

Other body fluids	09	4.1

Other	12	55.0


**Figure 1:**
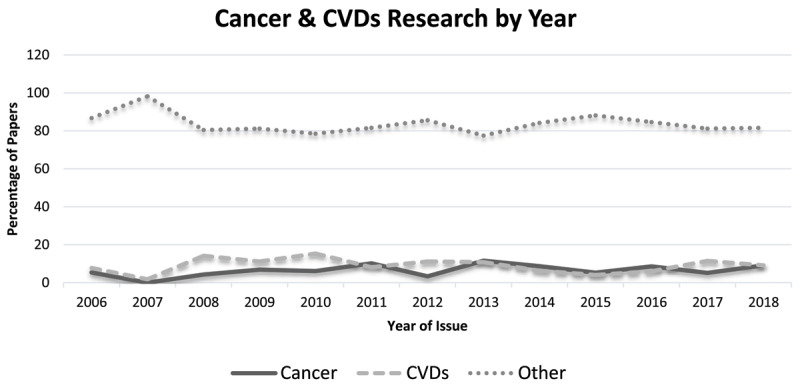
Persistent disparity in cancer and cardiovascular disease research for papers accepted at the CARPHA conferences from 2006 to 2018 (N = 1,512).

Research output for cancer and cardiovascular disease remained consistently low, as a proportion of the total research output in the Caribbean region through the entire period examined (***Figure 1***).

Cross-sectional studies were the most commonly utilized study design (43.8%), representing almost 50% of the studies in CVD research and approximately a quarter in cancer research (***Table 2***). Other common epidemiological study designs for these NCDs included cohort (12.9% cancer, 6.9% CVD) and case-control studies (10.9% cancer, 3.8% CVD). There were also a few qualitative studies (7.9% for cancer, 2.3% CVD). Noticeably no randomized controlled trials were presented for cancer or CVD in the meetings.

**Table 2:** Classification of methods for executing research accepted at CARPHA conferences from 2006 to 2018.


STUDY DESIGN	OVERALL N = 1498 (%)	CANCER N = 101 (%)	CVD N = 131 (%)	OTHER N = 1266 (%)

CROSS-SECTIONAL	658 (43.9)	25 (24.8)	60 (45.8)	573 (45.3)

Qualitative	90 (6.0)	8 (7.9)	3 (2.3)	79 (6.2)

Retrospective	88 (5.9)	13 (12.9)	9 (6.9)	66 (5.2)

Lab-based	77 (5.1)	5 (5.0)	2 (1.5)	70 (5.5)

Audit	75 (5.0)	5 (5.0)	7 (5.3)	63 (5.0)

Case series	69 (4.6)	7 (7.0)	5 (3.8)	57 (4.5)

Prospective	62 (4.1)	1 (1.0)	12 (9.2)	49 (3.9)

Case-control	43 (2.9)	11 (10.9)	5 (3.8)	28 (2.2)

Surveillance	44 (2.9)	2 (2.0)	2 (1.5)	39 (3.1)

Program evaluation	39 (2.6)	2 (2.0)	0 (0.0)	37 (2.9)

Intervention	28 (1.9)	0 (0.0)	3 (2.3)	25 (2.0)

Environmental	12 (0.8)	0 (0.0)	0 (0.0)	12 (1.0)

Other*	213 (14.2)	22 (21.8)	23 (17.6)	168 (13.3)


* This figure contains other methods that were too small as standalone e.g. animal study, clinical trials, outbreak investigation, literature review, systematic review, registry based, metabolic, validation, reliability, randomised, health services, modelling, cost benefit analysis etc.

A study design was not explicitly stated in over a third (39%) of all abstracts (34.7% for cancer research and 38.2% for CVD). There were also several discrepancies in the stated research design and the methods reported in the abstract in about a fifth of the cancer and CVD abstracts (***Table 3***).

**Table 3:** Percentage of cancer and cardiovascular diseases research accepted at the CARPHA conferences from 2006 to 2018 (N = 1,502).


AGREEMENT BETWEEN WRITTEN STUDY DESIGN & STEPS TAKEN	CANCER N (%)	CVD N (%)	OTHER N (%)

Agreement	43 (42.6)	53 (40.5)	482 (38.0)

Disagreement	23 (22.8)	28 (21.4)	280 (22.1)

Not Stated	35 (34.7)	50 (38.2)	508 (40.0)


## Discussion

Research on cancer and CVD remains underrepresented considering their contribution to mortality and morbidity in the Caribbean. This review corroborates claims that countries in the region are not fully engaged in research that is critical for providing locally relevant data to respond to the high burden of cancer and CVDs [9,10,11,12].

Most research abstracts originated from universities. This is consistent with the literature as universities are one of the main drivers of scientific research in developing countries as they build human capital and drive innovation [13]. However, despite the declarations and initiatives for the reduction in NCDs taken by governments at the national level, we were unable to quantify whether additional funding for research has been provided by governments to these institutions for research that can address this issue. The Ministry of Health and Wellness in Jamaica recently launched a research fund for investigators to conduct research that addresses NCD prevention and improves the health of persons living with these conditions [14]. We hope that this will serve as an impetus for other regional governments to do the same.

Financial resource constraints may explain the large numbers of cross-sectional studies undertaken in comparison to more sophisticated studies such as clinical trials, cohort and laboratory studies. However, the primary utilization of the cross-sectional design may also reflect limitations in institutional capacity to engage in more complex and time-consuming studies that are more logistically intensive, and require a high level of statistical and epidemiological expertise, and regulatory oversight.

Several errors in the abstracts suggest the need for greater support and guidance in the design of the study and preparation of research papers. Among the weaknesses highlighted were the high numbers of abstracts with limited information in the methodology specifically as it relates to the design of the study. This weakness could make it more difficult to publish findings at a global level and obtain external grant funding to undertake research requiring more advanced study designs.

It is important to note that the CARPHA meeting may not capture all the research output for the region. However, our findings are consistent with other studies reviewing data from other sources that also reported a low research output from the Caribbean in NCDs. For example, a recent PubMed review of breast cancer research output in the Caribbean identified a total of ninety-two (92) publications over the course of 42 years [15]. Most of these publications involved one or more Caribbean investigators as first or corresponding author and articles were published in the West Indian Medical Journal, a regional research journal published by the University of the West Indies [10].

The CARPHA conference has provided early career researchers with a showcase for their research ideas. This may be one factor impacting the quality of some of the work presented. It is possible that the region is producing higher quality research that are not being presented at the CARPHA conferences but at specialised international conferences, where there is potential for access to strategic partnerships, collaborations and funding. For example, investigators in cancer research may choose the African Caribbean Cancer Consortium (AC3) International Scientific and Training Conference as a forum for their work. The AC3 has held biannual meetings since 2007 which has primarily featured cancer research in the Caribbean [10,16]. The resulting collaborations have facilitated cancer publications, in other peer-reviewed Journals, that are led by Caribbean investigators, many of which include measurements of genetic and other biomarkers.

The region will however continue to struggle with medical research due to a relatively small number of persons with critical research expertise who are spread over a large geographic area. While several countries continue to undertake research, most initiatives are limited to larger Caribbean territories where the University of the West Indies has a main campus. These sites, as currently organized, may have challenges providing other territories with research support to increase output in these locations. The Caribbean Institute for Health Research of the University of the West Indies has recently launched a Postgraduate Diploma in Health Research and Epidemiology to help develop a cadre of scientists within the region who can help support this research agenda [17]. This programme virtually delivered addresses issues of distance and time constraints due to work commitments that many early Caribbean based researchers face. It also provides them with better access to mentorship from more established Caribbean and international researchers.

In addition to the small number of trained researchers, the absence of well-established health data information systems, an environment that encourages and tangibly supports research and dedicated research funding in these countries may make pursuing these regional research partnerships challenging.

The National Institutes of Health has recognized the importance of building research capacity in LMICs, which currently face the greater burden from NCDs by funding a series of planning grants to help build research capacity [6,18]. This paper forms part of a larger Needs Assessment under a planning grant for the Caribbean to facilitate collaboration of LMIC with partners in high income countries (HIC) for knowledge and expertise transfers [18]. This study’s findings will facilitate the construction of a road map for the establishment of a Caribbean Regional Center for Research Excellence (RCRE) with a focus on cancer and CVD. The vision of the RCRE is to facilitate development in the areas of research methodology/statistical support and laboratory/biomarker services. Provision is also being made to support community engagement activities that will enable people to be more involved in research, enhance research capacity and increase utilization of research findings. We trust that this will form part of a series of activities that will help to build the Caribbean’s regional capacity to address the NCD epidemic in a meaningful way.

## Additional File

The additional file for this article can be found as follows:

10.5334/aogh.2886.s1 Review of Scientific Meeting Abstracts. Review of Caribbean Public Health Agency’s conference abstracts to identify gaps in the Caribbean’s NCD research output.
